# Role of fermented dairy products in the health benefits of a mediterranean diet

**DOI:** 10.1007/s40520-024-02721-x

**Published:** 2024-03-19

**Authors:** René Rizzoli, Emmanuel Biver

**Affiliations:** Service of Bone Diseases, Geneva University Hospitals and Faculty of Medicine, 1211 Geneva 14, Switzerland

**Keywords:** Nutrition, Bone mineral density, Fracture risk, Cardiovascular risk, Diabetes, Gut microbiota, Prebiotics, Probiotics

## Abstract

Mediterranean diet includes fermented dairy products like yogurt and cheese. These foods provide calcium, phosphorus, fat, carbohydrates and protein, all nutrients influencing various systems including bone, cardiovascular system, intermediary metabolism, cancer, central nervous system, and inflammation. In addition, they contain prebiotics and provide probiotics which are capable of modifiying microbiota composition and metabolism, potentially acting also indirectly on the various systems. A large body of evidence indicates that fermented dairy products consumption significantly contributes to the beneficial effects of a Mediterranean diet on various systems’ health.

## Introduction

Fermented dairy products (FDP), particularly yogurts and cheese, have been used since 1000s of years to preserve milk, to make it more transportable, less perishable, readily available and more digestible, because of lactose breakdown during the fermentation process. This processing of milk was an important development in early agriculture, which can be dated back to the sixth millennium BC in Northern Europe [1]. Dairy products were ingested since 8000 years as shown by milk protein present in dental calculi of skeleton found in Africa [2].

Mediterranean diet (MedDiet) is associated with reduced risk of cardiovascular diseases, type 2 diabetes, cancer, inflammation, fracture and neurodegenerative disorders [3]. The question araises whether FDP, which are part of a MedDiet, contribute to the risk reduction of disorders affecting these various systems. At the beginning of the twentieth century, the Nobel Prize winner Yllia Metchnikoff has hypothesized that health could be improved, senility delayed and longevity prolonged by modifying gut microbiota (GM) through the ingestion of host-friendly bacteria like lactobacilli found in yogurt [4].

## Age-associated bone loss and fracture risk

A meta-analysis of observational studies including 13,209 participants showed that a greater adherence to a MedDiet was associated with a positive linear relationship with total hip and trochanter BMD [5]. This observation is in agreement with a previous meta-analysis of several observational studies having demonstrated a lower hip fracture risk in people adherent to a MedDiet [6].

**Fig. 1:**
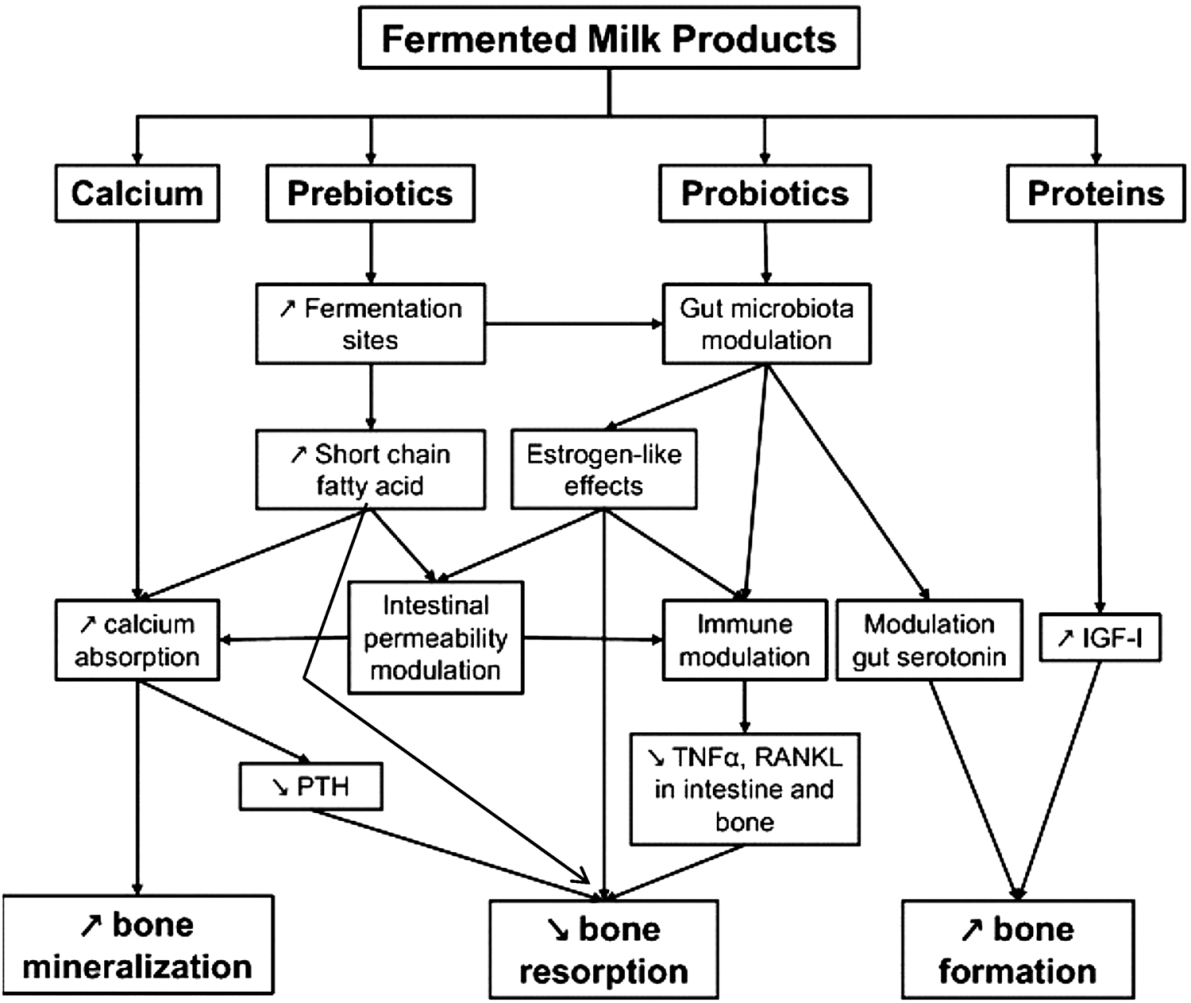
Influence of fermented dairy products on bone metabolism. Adapted from [7] with permission from the publisher

In adults, consumption of fermented dairy products attenuates age-related bone loss [7]. In a cross-sectional study in home dwelling subjects older than 60 years, yoghurts ingestion was associated with higher bone mineral mass and better muscle function [8]. For one serving of yoghurt per day, the risk of osteoporosis was 40 and 50% lower in women and men, respectively. In 65-year old healthy women, peripheral skeleton cortical bone loss was inversely correlated to yoghurt intake frequency [9]. Short-term intervention trials have shown that yoghurt or cheese consumption reduced PTH and biochemical markers of bone resorption, without affecting bone formation markers (for review [7, 10]) A number of controlled intervention trials have been conducted in adults testing the effects of FDP consumption on markers of bone activity. Consumption of a vitamin D and calcium-fortified soft cheese by healthy postmenopausal women increased protein intake, reduced the serum concentration of bone resorption biomarkers (TRAP 5b and CTX), and increased serum IGF-I, compatible with a nutrition-induced reduction in postmenopausal bone turnover rate [11]. Similar findings were found in studies in elderly women using soft cheese or yogurt [12, 13]. Bedtime consumption of fermented milk reduced nocturnal bone resorption [14]. Three servings a day of fortified milk or yogurt for 12 months induced more favorable changes in biochemical indexes of bone metabolism and bone mineral density than can be induced by calcium supplementation alone in postmenopausal women [15]. In a 20-year follow-up of 61,433 women, the risk of hip fracture was 0.70 and 0.64 for consumers of 400 g/day of yogurts or fermented milk, and of cheese, respectively [16]. For each serving (200 g of yogurt or 20 g of cheese), hip fracture risk was reduced by 10–15%. Several recent meta-analyses have addressed the question of fracture risk, particularly hip fracture, in relation with yogurt and cheese consumption (Table 1). In one of them, combining 3 cohorts and including 102,819 subjects, yoghurt consumption was associated with a 24% reduction in hip fracture risk [10]. The mechanisms of FDP influencing fracture risk are illustrated in Fig. 1.

**Table 1:** Fracture risk according to consumption of fermented dairy products (highest vs lowest) in meta-analyses (adapted from Biver [70])

	Yoghurt		Cheese	
	Number of studies	Risk (CI 95%)	Number of studies	Risk (CI 95%)
Bian et al. [71]				
Hip fracture	3	RR = 0.75 (0.66, 0.86)*	*3*	RR = 0.68 (0.61, 0.77)*
Hip fracture	1	OR = 0.77 (0.39, 1.52)	3	OR = 0.77 (0.53, 1.11)
Matia-Martin et al. [72]				
All fractures	2	HR = 0.92 (0.87, 0.98)*	*2*	HR = 0.89 (0.81, 0.98)*
Hip fracture	5	HR = 0.87 (0.71, 1.05)	4	HR = 0.80 (0.62, 1.03)
Vertebral fracture	1	HR = 1.18 (0.59, 2.39)	1	HR = 0.65 (0.33, 1.27)
Hidayat et al. [73]				
Hip fracture	4	RR = 0.78 (0.68, 0.90)*	4	RR = 0.85 (0.66, 1.08)
Ong et al. [10]				
Hip fracture	3	RR = 0.76 (0.63, 0.80)*	2	RR = 0.89 (0.73, 1.10)
Zhang et al. [74]				
All fractures			7	RR = 0.90 (0.86, 0.95)*
Hip fracture			7	RR = 0.86 (0.72, 1.04)

RR: Relative Risk (cohort studies); HR: Hazard Ratio (cohort studies); OR; Odds Ratio (case–control studies)

*Statistically significant

## Cardiovascular risk

Adherence to a MedDiet is associated with a 10% reduction in mortality or incident cardiovascular disease for each 2-point increase in adherence score, as shown in a 2010 meta-analysis [17]. The consumption of low fat instead of full fat dairy products to prevent cardiovascular disorders has been recommended in several guidelines [18, 19]. However, in a large prospective cohort study conducted in 21 countries and comprising 136,384 individuals, in whom 10,567 events were recorded, a greater dairy consumption was associated with a lower risk of cardiovascular disase and a lower mortality [20]. An overview of systematic reviews and meta-analyses of observational studies has concluded that consumption of total dairy products, either plain or with low fat content, did not adversely influence the risk of cardiovascular disorders [21]. More recently, a systematic review and meta-analysis of prospective cohorts studies has concluded that total dairies consumption was associated with a lower risk of coronary heart disease, hypertension, and stroke when individuals with the highest dairy intake were compared to those with the lowest [22].

Regarding more specifically FDP, a dose–response meta-analysis of prospective cohort studies has shown a 2% reduction of cardiovascular disease and all-cause mortality for an increased intake of 20 g/day of cheese, sour milk or yogurt [23]. These observations are in agreement with more recent data obtained in large prospective cohorts. In the 409,885 participants in the Pan-European EPIC cohort, 7198 coronary heart disease events occurred over a 12.5-year follow-up period. Increased consumption of yogurt and cheese was associated with reduced risk, while total dairy was not [24]. In the Alpha Omega cohort in the Netherlands, yogurt consumption was inversely related to cardiovascular mortality and all-cause mortality [25]. Finally, replacing milk by whole-fat yogurt products or cheese reduced myocardial infarction risk, as shown in the Danish Diet, Cancer and Health cohort [26]. Benefits of yogurt consumption on cardiovascular risk were also demonstrated in hypertensive patients [27]. Indeed, in 55,898 women of the Nurses’ Health Study and 18,232 men of the Health Profesionnals Follow-Up study, all with prevalent hypertension, ≥ 2 servings/week of yogurt compared to < 1/month lowered the risk of myocardial infarction or stroke by 17% and 21% in women and men, respectively [27]. A potential mechanism to account for these effects has to be sought in the so-called matrix effect of dairies [28]. For instance, a randomized controlled trial compared 120 g/day of full fat cheddar cheese to the same amount of fat and calcium achieved by 49 g of butter and of calcium salts over a 6-week intervention period. The cheese group but not the butter group experienced a reduction in total cholesterol and LDL [29]. In the MedDairy randomized cross-over controlled trial, supplementing a MedDiet with additional dairy foods to increase calcium intake resulted into a decrease in systolic and diastolic blood pressure, an increase in HDL cholesterol and a decrease in triglycerides level [30]. In long-term follow-up up to 30 years, combining 3 large cohorts (Nurses’ Health study I and II, *n* = 69,298 and 84,368 women, respectively, and Health Profesionnals Follow-up study, *n* = 30,512 men) showed that ≥5 servings of yogurt per week compared to <1 serving/month, was associated with a 16% reduction in the risk of incident high blood pressure [31]. For cheese (1–4 servings/day vs <1/week), the risk of high blood pressure was lower by 6%. These reductions remained significant after controlling for BMI. In 11,377 women and men aged between 40 and 99 participating in a population-based study, cheese intake was positively associated with HDL-C and inversely associated with LDL-C and triglycerides among non-users of cholesterol-lowering drugs [32]. Total intake of FDP was inversely associated with triglycerides levels while no associations were found for yogurt intake.

Altogether, these studies indicate that intake of FDP including fermented milk, yogurt and cheese, are associated with a lower cardiovascular risk [33]. Based on these evidences, recommendation has been made to include dairy products, especially FDP, like yogurt and cheese, in a healthy diet [34].

## Diabetes risk

Large body of evidence indicates that a MedDiet offers many benefits to patients with type 2 diabetes (T2D), both in the prevention of the disease and in its management such as in glycemic control [35–37]. This statement is based on epidemiological studies and on the results of a randomized controlled trial. In a subgroup of a large randomized controlled trial, in which adherence to a MedDiet enriched in extravirgin olive oil resulted in a 40% lower risk of T2D as compared with a control low-fat diet [38].

A systematic review and meta-analysis of randomized controlled trials concluded that increased dairy intake resulted in a reduction in fat mass, a gain in lean mass and a reduction of waist circumference as compared with controls [39].

A meta-analysis of five studies on changes in body weight per serving of dairy did not show any difference for whole-fat dairy and low fat dairy. However, there was an inverse association between changes in body weight for each yogurt serving increase, whereas each serving increase of cheese was positively associated. Furthermore, the highest dairy intake category was associated with lower risk of abdominal obesity and of overweight compared to the lowest intake category [40]. Thus, yogurt appears to play a role in weight managment [41].

In the Women Health Initiative study, in which 82,076 women were followed for 8 years, 3946 cases of incident treated diabetes occurred (annual incidence, 0.73%; cumulative incidence, 4.8%) [42]. After multivariable adjustments, low-fat dairy product intake was inversely correlated with the risk of T2D. Relative risk was around 0.6 in the upper quintile (median servings/day: 2.8) compared with the lowest quintile (0.05 serving/day). The inverse relationship was even more pronounced in women with a higher BMI. Highyogurt consumption (≥2/week vs <1/month) was associated with a lower T2D risk (relative risk 0.44), whereas there was no relationship between high-fat dairy products consumption and T2D [42]. The Malmö Diet and Cancer Cohort study showed a 23% reduction in T2D risk in 26,930 subjects followed for 14 years in the group with higher total, non-low fat dairy group [43]. The reduction was 20% for those consuming at least 180 g/day of yogurt. The association between long-term changes in dairy products intake and incident T2D was tested in three large US cohorts of women and men [44]. These cohorts were the Nurses’ Health Study I and II (76,531 and 81,597 women, repectively) and Health Professionals Follow-up Study (34,224 men), comprising altogether 2,783,210 person-year. Decreasing total dairy intake by > 1 serving/day during 4 years led to a 11% higher risk of T2D in the subsequent 4 years. In contrast, increasing yogurt consumption by >0.5 serving/day was associated with a 11% lower T2D risk, whereas increasing cheese intake by the same amount of servings led to a 9% increase in T2D risk. Substituting cheese by yogurt reduced the risk by 16% [44].

The relationship between the risk of T2D and the consumption of FDP has been addressed in numerous systematic reviews and meta-analyses [45–50]. All of them have concluded that increasing yogurt intake was associated with a lower risk of T2D. In the most recent one [50], 15 studies were included, comprising a total of 485,992 participants with 20,207 incident diabetes. Overall, a decreased diabetes risk was found to be associated with higher intake of FDP [odds ratio (OR) 0.925]. In a subgroup analysis, higher yogurt consumption was associated with lower T2D risk (OR 0.828). Thus, intake of FDP was associated with decreased T2D risk, and the effect appeared to be dose-dependent [50].

## Cancer risk

A recent narrative review on MedDiet for cancer prevention has concluded to an inverse relationship, or at least a neutral association, between the diet and risk of most types of cancer, with the limitation that mostly observational studies were analysed [51]. A meta-analysis including 117 studies with 3,202,496 participants has demonstrated a reduction of 13% for cancer mortality (18 studies), of 25% for all-cause mortality among cancer survivors (8 studies), of 6% for breast (23 studies), of 17% for colo-rectal (17 studies), of 44% for head and neck (9 studies), of 16% for respiratory (5 studies), of 30% for gastric (7 studies), 13% for bladder (4 studies), and of 36% for liver cancers (4 studies) [52]. The conclusion was that the highest adherence to a MedDiet was associated to a lower risk of cancer mortality and of all-cause mortality in cancer survivors, as well as a lower risk of cancer in some types of tumours, including colo-rectal cancer.

In the Italian part of the EPIC study, comprising 45,241 individuals, the consumption of yogurts in the highest tertile (85 and 98 g/day in men and women, respectively) was accompanied by a 35% lower risk of colo-rectal cancer in a multivariate model [53]. This study and six others, both cohort and case–control studies, were analysed in a review assessing the association between yogurt consumption and colo-rectal cancer [54]. Four papers reported higher yogurt consumption to be associated with lower risk of large intestine adenoma and of colo-rectal cancer, with OR varying between 0.47 and 0.90, when statistically significant.

## Neurodegenerative disorders

A systematic review and meta-analysis has evaluated the association between adherence to a MedDiet and mild cognitive impairment (MCI) or Alzheimer’s diseases (AD) [55]. In five studies which met eligibility criteria, higher adherence to the MedDiet was associated with a reduced risk of MCI and AD. The subjects in the highest MedDiet tertile had a 33% lower risk of MCI or AD as compared to the lowest MedDiet score tertile. Among cognitively normal individuals, higher adherence to the MedDiet led to a 27% and 36% reduced risk of MCI and AD, respectively. A randomized controlled trial investigated the effects of an extravirgin olive oil- or nuts-supplemented MedDiet as compared to a low fat diet control group (subgroup of the PREDIMED trial) over a median 4.1-year period [56]. Decrease from baseline of the global cognition composite test in the controls was blunted in the MedDiet group.

Regarding FDP, a 2-year follow-up of the PREDIMED-Plus study failed to detect any association between low fat milk, yogurt, cheese or FDP consumption, and changes in cognitive performance [57]. The results on the relationship between FDP consumption and cognitive function were not fully consistent. Indeed, in the cross-sectional analysis of baseline data collected in the frame of the PREDIMED-Plus trial, higher intake of FDP was observed in participants with a lower mini-mental state examination (MMSE) score (OR 1.340) [58]. In contrast, in Dutch community-dwelling adults aged ≥ 65 years, logistic regression analyses of cross-sectional data indicated that a 30 g increase in Dutch cheese intake was associated with a 33% lower probability of poor information processing speed [59]. In the Canadian longitudinal study on aging, total dairy product, cheese, and low-fat dairy product intakes were positively associated with the executive function domain, and yogurt intake with the memory domain, independently of age, gender, education, and diet quality [60]. In a cross-sectional study conducted in Japanese community-dwelling older adults, cheese intake was inversely associated with lower cognitive function as assessed by MMSE score [61].

Brain activity has been shown to be modulated by FDP. In a 4-week intervention with yogurt containing various probiotics in healthy women, functional magnetic resonance imaging detected changes in activity of brain regions controlling central processing of emotion and sensation [62]. Altogether, it appears that FDP intakes would be rather favorable on cognitive function. However, yogurt consumers may have healthier dietary habits and healthier non-nutritional behaviour than non-consumers, compatible with the hypothesis that yogurt consumption may be a marker of healthy diet and lifestyle.

## Fermented dairy products: possible mechanisms

The largest number of cells (10E^14^) within the human body are located within the intestinal tract. These organisms are collectively called the GM. They mostly refer to the large intestine content, but all parts of the GI tract are colonized with an increase in micro-organism concentration from the duodenum to the distal colon. Some studies have found that a MedDiet could modulate GM composition and function [63]. However, this systematic review indicated a lack of consistency for MedDiet modifying GM composition and metabolism. Such an inconsistency could be related to the heterogeneity of the population studied, the characterization of the MedDiet, variable duration and different analytical methods [63],

### Prebiotics

Prebiotics are non-digestible fiber compounds that pass undigested the upper part of the gastro-intestinal tract, and stimulate the growth and/or activity bacteriae that colonize the large bowel by acting as substrate for them [64]. Prebiotics refer to galactooligosaccharides (GOS), inulin, resistant starch, polydextrose, fructooligosaccharides, xylooligosaccharides, and lactulose. They ressemble oligosaccharides naturally present in human milk. Indeed, human milk contains various glycans with prebiotic properties contributing to infant immune system developmen [65]. The prebiotics inulin, which is derived from chicory roots, can be added to yogurt to increase the density of the matrix. Their mode of action implies the fermentation of fibers within the large intestine leading to the production of short chain fatty acids (SCFA) such as acetate, propionate, valerate, isovalerate, or butyrate and isobutyrate. SCFA have a large variety of effects in various systems [66].

### Probiotics

A way to modify microbiota is to directly provide some bacteriae to the GI tract, ie., probiotics [64]. Probiotics are live micro-organisms which, when administered in adequate amounts, confer a health benefit to the host. By adequate, one means an amount able to trigger the targetted effect. The concentration is around 10e^7^ to 10e^8^ probiotic bacteriae per gram, with serving size around 100 to 200 mg. Various species are provided as probiotics, such as Lactobaccilli, Bifidobacteriae, Escherichia, Enterococcus and Bacillus subtilis. Yeast like Saccharomyces have been used too. Probiotics are availabe in the form of yogurt, milk based foods, powder, capsules or solutions like ice cream or beer. One yogurt serving contains about 10 million bacteriae (*Lactobacillus bulgaricus et Streptococcus thermophilus*).

In human, the main source of probiotics is FDP. It has been reported that yogurt consumers had lower level of Enterobacteriaceae and higher beta-galactosidase activity, the latter and Bifidobacterium population being positively correlated to the amount of fermented products ingested [67].

A major problem with probiotics administration is certainly that a sufficient amount of bacteriae is ingested to modify GM composition. Indeed, in adult monozygotic tweens, two servings a day of FDP containing five different species of bacteriae, did not modify large intestine bacterial species composition. In contrast, when the same FDP were given by gavage to gnotobiotic mice, there was a rapid change (in less than 24 h) in microbiome encoded enzyms involved in carbohydrate metabolism [68].

An example of combining pre- and probiotics, reminiscent of a MedDiet, is provided by the results of a population-based large cohort study. In this study, the combination of fruits and vegetables, ie., prebiotics, with fermented milk (yogurt or soured milk), ie., probiotics, was associated with lower rates of hip fracture in high consumers of fermented milk and servings/day of fruits and vegetables, compared with low consumption of both fruit and vegetables and fermented milk [69].

## Conclusion

Through different mechanisms involving intakes of key nutrients such as calcium, phosphorus, fat, carbohydrates and protein, as well as pre- and probiotics, FDP consumption like yogurts or cheese, which are part of MedDiet, positively influence bone growth and bone homeostasis, cardiovascular health, cancer mortality, T2D risk and possibly cognitive function. Changes in GM composition and function may be implicated in these phenomena. The various effects observed in FDP consumers ressemble those observed in subjects adherent to a MedDiet, indicating that fermented dairy products may contribute to the health benefits associated with a Mediterranean diet.
